# Do mental wellbeing and emotional eating influence BMI similarly or differently? Evidence from a sample of Peruvian adolescents

**DOI:** 10.3389/fpubh.2025.1564656

**Published:** 2025-04-24

**Authors:** Keisler Cuyan-Zumaeta, David Javier-Aliaga, Mery Rodríguez-Vásquez, Jacksaint Saintila

**Affiliations:** ^1^Facultad de Ciencias de la Salud, Universidad Peruana Unión, Lima, Peru; ^2^Research Group for Nutrition and Healthy Behaviors, Universidad Señor de Sipán, Chiclayo, Peru

**Keywords:** mental wellbeing, mental health, emotional eating, BMI, adolescents

## Abstract

**Background:**

The literature highlights that both mental wellbeing and emotional eating are closely related to BMI, but little is known about how these variables interact similarly or differently. Therefore, the aim of this study was to determine the predictive role of mental wellbeing and emotional eating on body mass index (BMI) in Peruvian adolescents.

**Methods:**

This research used a non-experimental and predictive design. The sample consisted of 270 students aged 12–18 years from a public educational institution in the province of Tocache, Peru. The sample was selected through non-probability purposive sampling. The study employed the Mental Health Inventory (R-MHI-5), the Emotional Eating Scale (EES), and BMI was calculated using Quetelet’s formula.

**Results:**

Correlations revealed that mental wellbeing was negatively associated with BMI (*r* = −0.277, *p* < 0.001, 95% CI = [−0.384, −0.163]), whereas emotional eating was positively associated with BMI (*r* = 0.274, *p* < 0.001, 95% CI = [0.160, 0.381]). In the multiple regression analysis, Model 2 emerged as the most suitable (adjusted R^2^ = 0.112, *F* = 17.953, *p* < 0.001, BIC = 1,318), explaining 11.2% of the variance in BMI. Standardized coefficients indicated that mental wellbeing had a significant negative effect on BMI (*β* = −0.217, 95% CI = [−0.3353, −0.0997], *p* < 0.001), while emotional eating had a significant positive effect (*β* = 0.213, 95% CI = [0.0952, 0.3308], *p* < 0.001). Moreover, both coefficients showed similar magnitudes.

**Conclusion:**

The findings of this study confirm that mental wellbeing and emotional eating exert opposing yet similarly sized influences on BMI in Peruvian adolescents. These results underscore the importance of addressing both variables equitably in interventions aimed at improving adolescents’ nutritional status.

## Introduction

The high prevalence of inadequate Body Mass Index (BMI) among adolescents is a growing concern in public health and nutrition (1). According to the latest report from the World Health Organization (WHO), 8% of children and adolescents aged 5–19 worldwide are classified as obese (1). In Peru, two studies conducted in Lima revealed that 14 and 15.9% of adolescents are obese (2, 3). Moreover, inadequate BMI has significant psychological and physical effects on adolescents. Psychologically, it is associated with issues such as low self-esteem, depression, anxiety, and behavioral disorders (4). Physically, overweight and obesity increase the risk of cardiovascular problems and long-term non-communicable diseases (5).

Emotional eating is a significant factor contributing to inadequate BMI levels among adolescents. According to a representative study conducted in the United States in 2024, 30% of American adolescents reported eating in response to negative emotions, indicative of emotional eating (6). Furthermore, emotional eating in adolescents is associated with physical, psychological, and social consequences (7–11). Physically, it is linked to an increased risk of obesity and cardiovascular diseases due to the consumption of calorie-dense, nutrient-poor foods. Psychologically, it exacerbates depressive symptoms and contributes to the development of eating disorders, such as binge eating, creating a negative cycle of body dissatisfaction and mental health issues. Socially, poor emotional regulation and family environment play a role in fostering emotional eating, impacting interpersonal relationships and overall wellbeing. Finally, scientific literature has consistently demonstrated an association between emotional eating and BMI (12).

Mental wellbeing during adolescence is a public health issue and a key determinant of obesity and overweight in this age group (4, 13, 14). The World Health Organization (WHO) reported that one in seven adolescent’s aged 10–19 worldwide experiences mental wellbeing challenges (15). Moreover, mental wellbeing significantly impacts social, emotional, physical, and educational health outcomes (16). In the educational domain, mental wellbeing issues among adolescents can lead to poor school adaptation, concentration problems, and low academic performance (17). Psychologically, these challenges may result in anxiety, depression, psychological stress, and even suicide (18). Socially, they are associated with school dropout or expulsion and problematic peer relationships, while physically, they are linked to an increased rate of risky health behaviors, such as substance use (17). Finally, studies have demonstrated a relationship between mental wellbeing and BMI in adolescents (4).

Several studies have demonstrated the interaction between mental wellbeing and Body Mass Index (BMI) in adolescents (4, 14, 19–21). Similarly, other research has highlighted the relationship between emotional eating and BMI in this age group (8, 10, 12). However, there is a lack of studies investigating the combined impact of mental wellbeing and emotional eating on BMI. Considering the aforementioned points and the scarcity of research exploring BMI prediction based on independent variables such as emotional eating and mental wellbeing, this study seeks to provide a significant contribution to this field.

This research is justified by its contribution to understanding the interplay between mental wellbeing, emotional eating, and BMI in a sample of adolescents from northern Peru. Understanding these dynamics is essential for designing strategies that promote better physical and mental health during this critical developmental stage. Additionally, the study will have a social impact by providing valuable information to public and private organizations, enabling them to address issues such as obesity, emotional eating, and mental wellbeing from a public health and nutritional perspective. The data generated will also be instrumental for educational institutions in designing and implementing programs that target both mental wellbeing and emotional eating, aiming to reduce obesity within the educational context. Finally, the study’s findings will raise awareness of the importance of mental wellbeing and emotional eating in adolescents, empowering them to adopt healthier habits that contribute to maintaining an adequate BMI.

The general objective of this study is to determine the predictive role of mental wellbeing and emotional eating on BMI in a sample of Peruvian adolescents. Additionally, the specific objectives are as follows: (1) to determine the predictive role of mental wellbeing on BMI in a sample of Peruvian adolescents. (2) To analyze the predictive role of emotional eating on BMI in a sample of Peruvian adolescents. (3) To determine differences in mental wellbeing according to sex in a sample of Peruvian adolescents.

## Materials and methods

### Study design

This study employed a quantitative approach with a non-experimental, cross-sectional, and predictive design. The dependent variable was BMI, while the independent variables included mental wellbeing and emotional eating (22, 23).

### Sample

The non-probabilistic sample consisted of 270 adolescents from the second to fifth year of a public educational institution in the province of Tocache, Peru (22). Inclusion criteria included adolescents of both genders, aged 12–18 years, who provided informed assent and whose parents signed informed consent forms. Exclusion criteria included adolescents undergoing psychological treatment or with prior diagnoses of depression, anxiety, or chronic stress. The required sample size for a multiple regression model was estimated using the G*Power 3.1.9.7 statistical program (24). Calculations indicated that a sample of 107 individuals was sufficient to detect effects with a significance level of *α* = 0.05, statistical power of 0.95, a moderate effect size (f^2^ = 0.15), and two predictors. However, to ensure greater precision and robustness in the analyses, a larger sample size was selected for this study (*n* = 270). Data collection took place during the first 2 weeks of December 2024. The instruments were administered in person, with a response time of 6–8 min per adolescent, and anthropometric measurements (weight and height) were conducted within a 4-min range. These measurements were performed by two trained nutritionists, who underwent a standardization process prior to data collection, and a single measurement was taken. Participants’ responses were anonymous and voluntary.

Table 1 shows that the study participants consisted of 270 adolescents with an average age of 15.04 years (SD = 1.08). The gender distribution was balanced, with a slight majority of females (51.5%) compared to males (48.5%). Regarding grade level, nearly half of the participants were in third (40%) or fourth grade (35.9%) of secondary school, while approximately one in 10 were in second grade (10%), and a minority were in fifth grade (14.1%). Finally, concerning BMI, the majority had a healthy weight (68.9%), about a quarter were overweight (27%), and a minority were classified as obese (4.1%).

**Table 1:** Sociodemographic characteristics and BMI of the study sample (*n* = 270).

Characteristics	*n*	%
Age (M ± SD)	M = 15.04	SD = 1.08
Sex		
Male	131	48.5
Female	139	51.5
Grade level		
Second	27	10.0
Third	108	40.0
Fourth	97	35.9
Fifth	38	14.1
BMI		
Healthy weight	186	68.9
Overweight	73	27
Obesity	11	4.1

M, Mean; SD, Standard deviation; BMI, Body mass index.

### Instruments and measurement equipment

#### Emotional eating scale (EES)

The Emotional Eating Scale (EES) was developed by Garaulet et al. (25) to assess the influence of emotions on eating behaviors. This instrument consists of 10 items rated on a Likert scale ranging from “Never” (1) to “Always” (5). In the Spanish context, the average reliability value for the subscales of the EES was 0.7, indicating acceptable internal consistency. The temporal stability of the instrument was confirmed through test–retest reliability, with a correlation coefficient of 0.70. Additionally, the scale demonstrated good convergent validity with the Mindful Eating Questionnaire (MEQ). In this study, the instrument’s reliability was adequate, with a McDonald’s *ω* of 0.718. Therefore, the EES proves to have good validity and reliability.

#### Mental health inventory (R-MHI-5)

The Mental Health Inventory (R-MHI-5) was developed by Berwick et al. (26) and is a tool designed to assess mental wellbeing in adolescents and adults. The scale evaluates mental wellbeing through two dimensions: the first measures the presence of psychological wellbeing (items 2 and 4), while the second assesses the absence of psychological distress using reverse-scored items (items 1, 3, and 5). Both dimensions reflect the individual’s mood and overall wellbeing. The inventory consists of 5 items, with response options on a Likert scale ranging from “Never” (0), “Sometimes” (1), “Often” (2), to “Always” (3). The R-MHI-5 was validated by Rojas-Mendoza et al. (27). Confirmatory factor analysis results indicated that a two-factor correlated model demonstrated superior fit indices (CFI = 0.99; TLI = 0.99; SRMR = 0.04; RMSEA = 0.101) compared to a unidimensional model (CFI = 0.85; TLI = 0.71; SRMR = 0.23; RMSEA = 0.451). The dimensions showed adequate reliability: psychological wellbeing (*ω* = 0.88) and psychological distress (ω = 0.79). In this study, the instrument’s reliability was adequate, with a McDonald’s ω of 0.617.

#### BMI

The BMI of adolescents was assessed using anthropometric measurements (weight and height), along with age and sex (28–30). Weight was measured using the Tanita (Baby/Mon) digital scale, model 1,582, while height was determined using a standard stadiometer. Anthropometric measurements (weight and height) were conducted following a standardized process based on the WHO recommendations (31). The age and sex were collected through a sociodemographic questionnaire. Additionally, BMI categories were determined according to the age- and sex-specific cut-off points established by WHO (32). Adolescents were classified as underweight (< −2 SD), normal weight (−2 SD to +1 SD), overweight (> +1 SD to +2 SD), and obese (> +2 SD) based on the BMI-for-age z-scores.

#### Statistical techniques for data analysis

Data analysis was conducted using SPSS software (version 29) and RStudio (version 2023.12.0–0). Descriptive analysis included measures of central tendency, such as the mean and standard deviation. For inferential analysis, Pearson’s correlation coefficient, Student’s *t*-test, and multiple linear regression models were employed. Additionally, the Kolmogorov–Smirnov test was used to assess data normality. However, according to the Central Limit Theorem, which states that in samples larger than 30 participants, the sampling distribution tends to normality, ensuring the validity of statistical methods, normality of the variables is not a fundamental requirement for the application of parametric tests such as Student’s *t*-test, Pearson’s correlation, and linear regression, especially in studies with large sample sizes (33). Furthermore, Cohen’s d was calculated to interpret the effect size and the magnitude of observed differences. A significance level of 5% (0.05) was used to determine the statistical significance of correlations and the coefficients in the multiple linear regression model, with a 95% confidence interval.

## Results

The analysis of Table 2 reveals significant differences in mental wellbeing by sex, with males (M = 17.36, SD = 2.92) reporting higher levels than females (M = 16.65, SD = 2.51), accompanied by a small but meaningful effect size (*p* = 0.033, 95% CI = [0.057, 1.366], d = 0.2617). However, no significant differences in mental wellbeing were observed across age groups (*p* = 0.361, 95% CI = [−0.389, 1.063], d = 0.1232). For emotional eating, no significant differences were found by age (*p* = 0.753, 95% CI = [−2.233, 1.617], d = −0.0406) or sex (*p* = 0.051, 95% CI = [−0.359, 0.012], d = −0.2375), although females (M = 25.95, SD = 7.87) tended to score higher than males (M = 24.16, SD = 7.16), reflecting a small effect size. Finally, BMI showed no significant differences either by age (*p* = 0.469, 95% CI = [−1.019, 0.471], d = −0.0963) or by sex (*p* = 0.865, 95% CI = [−0.742, 0.624], d = −0.0207), with very similar averages across the analyzed groups. These findings indicate that mental wellbeing is the only variable with significant differences, particularly in relation to sex, while emotional eating and BMI remain consistent across the sociodemographic groups studied.

**Table 2:** Descriptive and comparative analysis of mental wellbeing, emotional eating, and BMI by sociodemographic variables.

Sociodemographic variables	Mental wellbeing		Emotional eating		BMI	
	M (SD)	*p,* CI, *d*	M (SD)	*p,* CI, *d*	M (SD)	*p,* CI, *d*
Age^a^						
12–14 years	17.22 (2.86)	0.361 [−0.389, 1.063] 0.1232	24.87 (7.32)	0.753 [−2.233, 1.617] −0.0406	22.01 (2.91)	0.469 [−1.019, 0.471] −0.0963
15 a 18 years	16.89 (2.67)		25.18 (7.70)		22.28 (2.82)	
Sex^a^						
Male	17.36 (2.92)	0.033* [0.057, 1.366] 0.2617	24.16 (7.16)	0.051 [−0.359, 0.012] −0.2375	22.17 (2.85)	0.865 [−0.742, 0.624] −0.0207
Female	16.65 (2.51)		25.95 (7.87)		22.22 (2.85)	

CI, Confidence Interval; ^a^Student’s *t*-test; d, Cohen’s d. BMI, body mass index. *p < 0.05. ^a^ indicates the statistical test used.

Table 3 shows the correlations between mental wellbeing, emotional eating, and BMI in adolescents. The results indicate a significant negative correlation between mental wellbeing and BMI (*r* = −0.277, *p* < 0.001, 95% CI = [−0.384, −0.163]), suggesting that higher mental wellbeing is associated with lower BMI in adolescents. Conversely, emotional eating is positively correlated with BMI (*r* = 0.274, *p* < 0.001, 95% CI = [0.160, 0.381]), implying that a decrease in emotional eating is associated with a lower BMI.

**Table 3:** Correlation analysis between mental wellbeing, emotional eating, and BMI in adolescents.

Variables	Mental wellbeing	Emotional eating	BMI
Mental wellbeing	1		
Emotional eating	−0.279^***^ [−0.386, −0.165]	1	
BMI	−0.277^***^ [−0.384, −0.163]	0.274^***^ [0.160, 0.381]	1

^***^The relationship analysis was done using Pearson’s correlation coefficient; BMI, body mass index.

Table 4 presents the analysis of the multiple regression model, concluding that Model 2, compared to Model 1, is statistically significant both globally (adjusted R^2^ = 0.112, *F* = 17.953, *p* < 0.001) and in the individual coefficients of the predictive variables (*p* < 0.001). Additionally, the lower BIC value in Model 2 (BIC = 1,318) supports that this model is more suitable and efficient for predicting BMI in the studied population. Regarding the explanatory power of the predictors, the adjusted coefficient of determination (adjusted R^2^ = 0.112) indicates that mental wellbeing and emotional eating jointly explain 11.2% of the variability in adolescents’ BMI. The standardized coefficients show that mental wellbeing has a significant negative effect on BMI (*β* = −0.217, 95% CI = [−0.3353, −0.0997], *p* < 0.001), while emotional eating exerts a significant positive effect (*β* = 0.213, 95% CI = [0.0952, 0.3308], *p* < 0.001). Both coefficients have similar magnitudes. These findings indicate that better mental wellbeing is associated with a lower BMI, while higher emotional eating is linked to an increased BMI in the evaluated adolescents.

**Table 4:** Multiple regression model.

Model		Standardized coefficients		*t*	TOL	VIF	*p*
		*β*	CI				
1	(Constant)			7.724			< 0.001
	Mental wellbeing	−0.215	[−0.3352, −0.0958]	−3.544	0.896	1.116	< 0.001
	Emotional eating	0.219	[0.1007, 0.3377]	3.641	0.913	1.095	< 0.001
	Age	0.047	[−0.0674, 0.1618]	0.812	0.977	1.023	0.418
	Sex	−0.040	[−0.3106, 0.1490]	−0.693	0.970	1.031	0.489
2	(Constant)			17.952			< 0.001
	Mental wellbeing	−0.217	[−0.3353, −0.0997]	−3.635	0.922	1.084	< 0.001
	Emotional eating	0.213	[0.0952, 0.3308]	3.561	0.922	1.084	< 0.001

Dependent variable: body mass index (BMI); Model 1: R^2^ adjusted = 0.109, ANOVA F (F = 9.259, *p* < 0.001), BIC = 1,328; Model 2: R^2^ adjusted = 0.112, ANOVA F (F = 17.953, *p* < 0.001), BIC = 1,318. CI = Confidence interval.

In summary, the results show that, in descriptive and comparative terms, mental wellbeing exhibits significant differences by sex, being higher in males (M = 17.36, SD = 2.92) than in females (M = 16.65, SD = 2.51; *p* = 0.033, 95% CI = [0.057, 1.366], d = 0.2617). Correlation analyses reveal that mental wellbeing is negatively associated with BMI (*r* = −0.277, *p* < 0.001, 95% CI = [−0.384, −0.163]), while emotional eating is positively associated with BMI (*r* = 0.274, *p* < 0.001, 95% CI = [0.160, 0.381]). In the multiple regression analysis, Model 2 emerges as the most suitable (adjusted R^2^ = 0.112, *F* = 17.953, *p* < 0.001, BIC = 1,318), explaining 11.2% of the variability in BMI. The standardized coefficients indicate that mental wellbeing has a significant negative effect on BMI (*β* = −0.217, 95% CI = [−0.3353, −0.0997], *p* < 0.001), whereas emotional eating exerts a significant positive effect (*β* = 0.213, 95% CI = [0.0952, 0.3308], *p* < 0.001). Both coefficients show similar magnitudes.

## Discussion

The high prevalence of inadequate BMI in adolescents represents a growing public health concern. Globally, 8% of children and adolescents aged 5–19 are classified as obese (1). In Peru, studies conducted in Lima report obesity rates of 14 and 15.9% (2, 3). This issue leads to psychological effects, such as low self-esteem, depression, and anxiety (4), as well as physical consequences, including overweight, obesity, and an increased risk of cardiovascular and non-communicable diseases (5). Inadequate mental wellbeing and emotional eating are significant factors contributing to unhealthy BMI values in adolescents (4, 12). Within this context, the primary objective of the present study is to determine the relationship between mental wellbeing, emotional eating, and BMI in a sample of Peruvian adolescents.

The present study found a significant negative association between mental wellbeing and BMI (*r* = −0.277, *p* < 0.001, 95% CI = [−0.384, −0.163]). Additionally, multiple regression analysis (*β* = −0.217, 95% CI = [−0.3353, −0.0997], *p* < 0.001) revealed that mental wellbeing is a significant predictor of BMI, suggesting that adolescents with higher levels of mental wellbeing are more likely to have lower BMI. These findings align with previous research. For instance, Chen et al. (19), in a study involving over 1 million adolescents from Europe and North America, reported that BMI was correlated with mental wellbeing. Specifically, adolescents with low body weight, overweight, or obesity exhibited more psychosomatic symptoms compared to those with healthy weight. Similarly, Nauli et al. (14), identified a correlation between nutritional status and mental wellbeing scores among Islamic adolescents. Förster et al. (20) also demonstrated a significant negative association between BMI and scores on the psychological wellbeing scale. In the same vein, Beltrán-Garrayo et al. (21), in a prospective study examining the relationship between obesity and mental health disorders from childhood to adolescence, found that adolescents with obesity exhibited a higher prevalence of mental health disorders. Moreover, psychological comorbidities increased over a five-year period, and childhood obesity was associated with a higher risk of psychological diagnoses in adolescence.

Scientific literature has shown that mental wellbeing issues, such as depression, anxiety, and low self-esteem, are more prevalent in adolescents with obesity compared to their non-obese peers (34). Depression, in particular, is closely linked to obesity, as the latter is considered an inflammatory state associated with depressive disorders (35). Mental health disorders, such as depression, can trigger inflammatory processes that are related to metabolic alterations and increased body weight (36). Additionally, mental wellbeing disturbances in adolescents are associated with increased release of stress-related neurotransmitters, such as cortisol and norepinephrine (37). Elevated and sustained cortisol levels are linked to abdominal fat accumulation, insulin resistance, and the development of unhealthy eating patterns (38). This imbalance can lead to frequent consumption of energy-dense foods, such as junk food, contributing to a higher Body Mass Index (BMI), overweight, and obesity (39). Stress and poor mental wellbeing also affect leptin function, a key hormone in appetite regulation and energy balance, resulting in excessive food consumption (40). Moreover, impaired mental wellbeing is associated with unhealthy lifestyles, including sedentary behavior, sleep disturbances, and disorganized eating habits, all of which increase the risk of overweight and obesity (35). Finally, adolescent mental wellbeing, both in the short and long term, is closely tied to higher body weight and its consequences, including cardiovascular diseases, type 2 diabetes, and other related conditions (19, 41). This highlights the critical importance of addressing mental wellbeing as a fundamental component in the prevention and treatment of adolescent overweight and obesity.

On the other hand, this study also found a positive association between emotional eating and BMI (*r* = 0.274, *p* < 0.001, 95% CI = [0.160, 0.381]). Additionally, multiple regression analysis (*β* = 0.213, 95% CI = [0.0952, 0.3308], *p* < 0.001) revealed that emotional eating is a significant predictor of BMI, suggesting that adolescents with higher levels of emotional eating are more likely to have higher BMI. Similar results were reported by Bektas and Gürkan Kübrn (12), who demonstrated that adolescent BMI was positively correlated with emotional eating. Their study also showed that 14.1% of the variance in BMI levels was explained by emotional eating and self-efficacy in weight control. Likewise, Rachmawati et al. (10) found that emotional eating and the consumption of high-calorie snacks were associated with higher BMI, contributing to overweight in this population. Additionally, Shriver et al. (8) revealed that emotional eating was positively related to adiposity in late-stage adolescents.

Emotional eating is associated with increased BMI in adolescents due to several factors. First, eating in response to emotions rather than physical hunger activates the hypothalamic–pituitary–adrenal axis, increasing cortisol release, a hormone that stimulates appetite, particularly for sugar- and fat-rich foods (42). Second, consuming these foods triggers dopamine release in the brain, creating a pleasurable sensation that reinforces the habit of eating in response to negative emotions (43). Third, psychological factors such as low self-esteem, anxiety, and stress hinder healthy emotional regulation, promoting maladaptive eating patterns (44). Finally, emotional eating can contribute to overweight and obesity by establishing a cycle in which consuming calorie-dense foods as a coping strategy perpetuates energy imbalances, metabolic disruptions, and feelings of guilt, exacerbating the problem (45). This negative and cyclical dynamic underscores the need for further studies to deepen the understanding of these variables and the development of interventions targeting them to improve adolescents’ quality of life.

In this comparative study, significant differences in mental wellbeing were identified by sex, with males (M = 17.36, SD = 2.92) reporting higher levels than female adolescents (M = 16.65, SD = 2.51; *p* = 0.033, 95% CI [0.057, 1.366], d = 0.26). This indicates that, on average, males reported higher mental wellbeing compared to females, and this difference is statistically significant. The effect size (d = 0.26) suggests a small magnitude according to Cohen’s criteria, indicating that while the difference is significant, its practical impact may be limited. Several studies have reported significant sex differences in mental wellbeing among adolescents, with findings showing higher wellbeing levels in males compared to females (46–49). Females tend to be more emotionally expressive, which may lead to a greater perception and reporting of emotional distress (50). Additionally, males often use coping strategies focused on distraction or externalization, whereas females may lean toward rumination, increasing their vulnerability to mental wellbeing issues (45, 51). Furthermore, societal expectations and gender roles may place additional burdens on female adolescents, negatively impacting their mental wellbeing (52). These findings highlight the importance of considering sex differences in the development of targeted interventions to support adolescent mental wellbeing.

According to the adjusted R^2^ obtained from the multiple regression analysis (adjusted R^2^ = 0.112), 11.2% of the variability in Body Mass Index (BMI) is explained by mental wellbeing and emotional eating. This indicates that the remaining 88.8% of BMI variability is associated with other variables not included in the model. These variables may include various factors influencing BMI, such as familial, social, and physical aspects, among others. Therefore, the 11.2% variability in BMI can be attributed to the combined influence of mental wellbeing and emotional eating.

Additionally, the study’s results revealed that the standardized coefficients showed that mental wellbeing and emotional eating have similar effects on BMI (mental wellbeing: *β* = −0.217, 95% CI = [−0.3353, −0.0997], *p* < 0.001; emotional eating: *β* = 0.213, 95% CI = [0.0952, 0.3308], *p* < 0.001). This suggests that both variables contribute proportionally to the model, albeit in opposite directions. This finding implies that psychological factors, such as mental health, and behavioral factors, such as maladaptive eating behaviors, exert equivalent influence on BMI. This equivalence highlights the need to address both dimensions in an integrated manner within explanatory models and intervention designs. The relative balance of these factors underscores that psychological aspects, like mental wellbeing, can have as significant an impact as behavioral factors like emotional eating in weight regulation. This interplay emphasizes the importance of considering the interaction and synergy between these two factors to develop effective strategies for promoting holistic health in adolescents.

### Public health implications

According to the results of this study, the implications for the field of nutritional public health are significant. First, it is recommended that programs aimed at reducing Body Mass Index (BMI) in adolescents adopt an integrated approach that considers mental health. This can be achieved by implementing strategies and developing skills that promote adolescent mental wellbeing. Additionally, intervention plans should address emotional eating by emphasizing the importance of understanding the underlying reasons for eating—whether to satisfy a physical need or as a response to emotions. In this regard, it is essential to educate adolescents about the risks and implications of emotional eating, fostering greater awareness of their eating patterns and the impact these have on their physical and mental health. Second, public health interventions, particularly in nutritional public health, should adopt a holistic perspective. This means not limiting efforts solely to the physical aspect but also integrating the mental and emotional-behavioral components of adolescents. Such an integrated approach would more effectively address the multiple dimensions influencing BMI and the overall health of this age group, thereby promoting more sustainable and personalized interventions. Finally, the finding that mental wellbeing and emotional eating have a similar impact on adolescents’ BMI underscores the need for intervention programs to emphasize both constructs equally. Consequently, public health policies aimed at reducing BMI in this population must be multifaceted, integrating strategies that address not only physical factors but also mental and emotional-behavioral aspects. This holistic approach will enable the design of more comprehensive and effective interventions, promoting the overall wellbeing of adolescents.

### Limitations and future perspectives

The results of this study are significant and highly relevant, as previously mentioned and discussed. However, it is important to acknowledge certain limitations. First, the cross-sectional design does not allow for causal relationships to be established between BMI and the variables evaluated in adolescents, limiting the findings to correlations. Additionally, the non-probabilistic sample was restricted to adolescents from a specific geographic region in northern Peru, which limits the generalizability of the results to other populations with different sociodemographic and cultural characteristics. One of the limitations of this study is that variables such as diet, physical activity, or socioeconomic status, among others, were neither collected nor adjusted for, despite being key determinants of BMI. This may influence the results and should be interpreted within this context. This aspect could be considered as an objective for future studies. Finally, the use of self-reported instruments may be subject to social desirability bias and potential errors in participants’ perception or recall, which should be considered when interpreting the findings. In terms of future perspectives, it is recommended that research explore the variables considered in this study using alternative statistical approaches, such as mediation, moderation, or structural equation modeling. Moreover, it would be valuable to assess the dynamics between mental wellbeing and emotional eating through longitudinal studies to observe changes and patterns over time. Future studies should include more diverse samples, considering geographic, socioeconomic, and cultural origins, to examine whether these findings replicate in different contexts. Finally, incorporating additional explanatory factors of BMI, such as contextual and familial influences, is suggested to achieve a more comprehensive understanding of the factors affecting BMI in adolescents.

## Conclusion

The results of this study indicate that mental wellbeing and emotional eating exert opposing but equally significant influences on BMI in Peruvian adolescents. Higher mental wellbeing is associated with lower BMI, whereas higher emotional eating correlates with increased BMI. These findings highlight the need for interventions that integrate both psychological and behavioral factors to improve adolescents’ nutritional status and overall wellbeing. From a public health perspective, addressing mental wellbeing alongside dietary habits is essential for effective BMI management. Schools, healthcare institutions, and policymakers should implement strategies that promote mental wellbeing and equip adolescents with coping mechanisms to reduce emotional eating. Despite its contributions, this study has limitations. The cross-sectional design does not establish causality, and the non-probabilistic sample limits generalizability. Future research should employ longitudinal designs and incorporate additional factors such as physical activity and socioeconomic status to enhance understanding of BMI regulation in adolescents. In conclusion, mental wellbeing and emotional eating play a crucial role in BMI variations among adolescents. Their balanced yet opposite influence underscores the need for multidimensional interventions that address both mental health and eating behaviors to develop more effective public health strategies.

## Data Availability

The raw data supporting the conclusions of this article will be made available by the authors, without undue reservation.
